# Conductive Hydrogel Tapes for Tripolar EEG: A Promising Solution to Paste-Related Challenges

**DOI:** 10.3390/s24134222

**Published:** 2024-06-29

**Authors:** Cassidy Considine, Walter Besio

**Affiliations:** Department of Electrical, Computer and Biomedical Engineering, University of Rhode Island, Kingston, RI 02881, USA; considinec@uri.edu

**Keywords:** hydrogel, conductive tape, electroencephalography (EEG), tripolar concentric ring electrodes (TCREs), tripolar EEG (tEEG)

## Abstract

Electroencephalography (EEG) remains pivotal in neuroscience for its non-invasive exploration of brain activity, yet traditional electrodes are plagued with artifacts and the application of conductive paste poses practical challenges. Tripolar concentric ring electrode (TCRE) sensors used for EEG (tEEG) attenuate artifacts automatically, improving the signal quality. Hydrogel tapes offer a promising alternative to conductive paste, providing mess-free application and reliable electrode–skin contact in locations without hair. Since the electrodes of the TCRE sensors are only 1.0 mm apart, the impedance of the skin-to-electrode impedance-matching medium is critical. This study evaluates four hydrogel tapes’ efficacies in EEG electrode application, comparing impedance and alpha wave characteristics. Healthy adult participants underwent tEEG recordings using different tapes. The results highlight varying impedances and successful alpha wave detection despite increased tape-induced impedance. MATLAB’s EEGLab facilitated signal processing. This study underscores hydrogel tapes’ potential as a convenient and effective alternative to traditional paste, enriching tEEG research methodologies. Two of the conductive hydrogel tapes had significantly higher alpha wave power than the other tapes, but were never significantly lower.

## 1. Introduction

Electroencephalography (EEG) remains a fundamental tool in neuroscience, facilitating the non-invasive examination of brain activity with applications ranging from clinical diagnosis to cognitive research [1]. However, EEG suffers from poor spatial resolution due to the blurring effects primarily from different conductivities of the volume conductor [2]. To improve the spatial resolution, the surface Laplacian has been applied to EEG [2,3]. The surface Laplacian is a high-pass spatial filter which sharpens the blurred potential distribution on the surface [3] and produces an image proportional to the cortical potentials [4]. Further, artifact contamination is particularly problematic at the start of seizures, where muscle and movement artifacts obscure the site of seizure origin or even the seizure itself [5].

**Figure 1:**
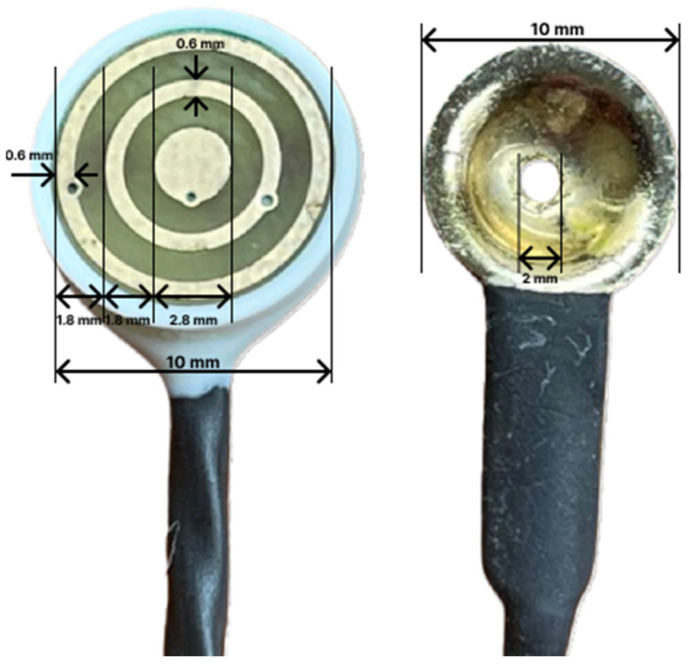
Tripolar Electrode on (**left**) and Disc electrode on the (**right**).

To overcome these drawbacks, Besio [6] developed the tripolar concentric ring electrode (TCRE) sensor and instrumentation to register tripolar EEG (tEEG). The distinctive TCRE design enables high-fidelity EEG recording that is an appreciable improvement over conventional disc electrodes. When taking bipolar differences from the closely spaced elements of the TCRE sensors, noise that is common to each element is automatically attenuated. The TCRE is directionally independent of global sources and highly focused on local activity due to its concentric configuration, which attenuates distant radial signals and artifacts by 100 dB one radius from the electrode [6]. Thus, a significant advantage of tEEG over EEG is that common artifacts such as muscle and ECG are attenuated in the recording [7]. Further, we have shown that the signal–noise ratio (SNR) of tEEG signals recorded with TCREs was 374% better than that of EEG [8]. For artifact suppression, unlike software-filtered data that may not represent the underlying biology, tEEG’s artifact suppression is faithful to the underlying neuronal activity. Finally, tEEG localizes more independent sources [6,8] and provides significantly better spatial resolution (about 10 times improvement over conventional EEG using the same electrode size) [9]. We have also found that TCREs can detect high-frequency activity prior to seizures that was not present in the conventional EEG that was recorded concurrently [10]. Further, we previously reported that the signals from the outer ring of the TCRE are equivalent to conventional EEG (eEEG) [11]. The TCREs and preamplifier (tInterface 20), necessary for their use, were obtained from CREmedical (East Greenwich, RI, USA) [9]. (See Figure 1).

Traditional EEG recordings typically involve the application of a conductive paste, such as the widely used Ten 20 paste (Weaver and Company, Aurora, CO, USA), to ensure optimal electrical contact between electrodes and the scalp. While effective, the use of paste presents several drawbacks, including impedance changing over time, inconvenience during the application, skin irritation, and a lengthy cleanup process post-recording. Further, since the electrodes of the TCRE sensors are so close together, 1.0 mm, if the paste is too conductive, there is not enough potential to register when the instrumentation takes the difference between the electrodes. Having a very high impedance between the electrodes of the TCRE sensor is optimal; however, if the impedance is too high, it attenuates ionic current flow from the scalp to the electrodes, decreasing the registered signals. Therefore, we must compromise the impedance between the electrodes of the TCRE sensor and the skin-to-electrode impedance. Further, different ionic current lengths, such as if the paste depth varies, could cause asymmetry with larger potential contributions from the shorter path.

To address these limitations, researchers have explored alternative electrode application methods, one of which involves combining hydrogels with electrodes. Although hydrogel electrodes are commonly used for electrocardiogram (ECG), they are seldom used for EEG other than for neonatal recordings where skin integrity is critical [12]. Hydrogel electrodes represent a relatively novel approach designed to enhance comfort during wear and streamline application processes. The hydrogel component of these electrodes serves a dual purpose: it aims to hydrate the stratum corneum, the outermost layer of the skin, while simultaneously improving conductivity and uniformity for a better signal quality [13]. Traditionally, in most hydrogel electrode research, the hydrogel is prepared separately and then affixed to an electrode substrate [14,15]. However, to deviate from conventional methods, our approach involved buying hydrogel tapes readily available in the market and affixing tripolar electrodes onto them. Manufacturing hydrogels can be a meticulous process prone to errors, variability, and inconsistencies with material properties, which could impact the quality of the recorded data [16]. The 10. 0 mm dia. TCRE typically can sit flat on most areas of the scalp. The commercially available hydrogels are of uniform thickness assuring the TCRE is parallel with the scalp surface. By using commercially available hydrogel tapes, it allowed for faster testing and enhanced reproducibility.

The focus of this study was to evaluate the efficacy of hydrogel tapes as an alternative to traditional paste for EEG electrode application. The specifications for the tapes are not disclosed by the manufacturer, so we also tried to characterize the tapes the best we could by measuring impedances. Specifically, we aim to assess the quality of the EEG recordings obtained using four different hydrogel tapes and determine which hydrogel presented as the best. 

In this manuscript, we present the results of our investigation into the use of hydrogel tapes for EEG recordings. We conducted multiple recording sessions with different participants using the four hydrogel tapes, comparing the impedances on the tapes and alpha wave characteristics. Through this comparative analysis, we aim to provide valuable insights into the feasibility and utility of hydrogel tapes in EEG research.

## 2. Materials and Methods

### 2.1. Subjects

The participants in this study were recruited on a voluntary basis from the University of Rhode Island. The inclusion criteria encompassed healthy adults. The study was conducted in accordance with the Declaration of Helsinki and approved by the Institutional Review Board (or Ethics Committee) of the University of Rhode Island (protocol IRB2122-185, 22 September 2022). Informed consent was obtained from all the subjects involved in the study. The demographic of the participants was 6 women and 4 men; all of whom were in their early 20s.

### 2.2. Hydrogel Tapes

Four hydrogel tapes were used for this study. BTHG-250X Hydrogel Tape was collected from Breachers Tape (Lake Zurich, IL, USA) and the KM40A, KM40C, and KM50L variants were obtained from Katecho (Des Moines, IA, USA). The BTHG-250X tape is a conductive hydrogel and demonstrated good longevity, making it suitable for our purposes. KM40C, KM40A, and KM50L are standardized medical hydrogel tapes that have undergone rigorous testing and validation procedures at Katecho. Throughout the study, all the tapes were stored in a metalized bag under ambient room temperature conditions to maintain their integrity and properties.

### 2.3. Tape Impedance

Ten random locations were chosen on each of the four hydrogel tapes for impedance testing. A TCRE was applied to these locations and the impedance was measured from the outer ring to the inner ring and from the middle ring to the inner ring using a 1089 Checktrode impedance meter (UFI, Morro Bay, CA, USA). The ten measurements from each tape were then averaged (Table 1).

### 2.4. Disc Electrodes on Head

In preparation for the EEG recording, a targeted area approximately 1.0 inch in diameter along the midline of the forehead was cleaned and treated with NUPREP. Subsequently, a conventional disc electrode, serving as the reference/ground electrode, was affixed to this spot using Ten 20 paste. Another disc electrode was positioned approximately an inch above the external occipital protuberance, also secured with Ten 20 paste. An impedance meter (1089NP Checktrode EEG, UFI) was then used to measure between both electrodes to verify that low skin-to-electrode impedance was achieved. The second disc electrode was used as a check for the presence of alpha waves as it is normal that some people do not have them.

### 2.5. TCREs and Hydrogel Tape Application on Head

**Figure 2:**
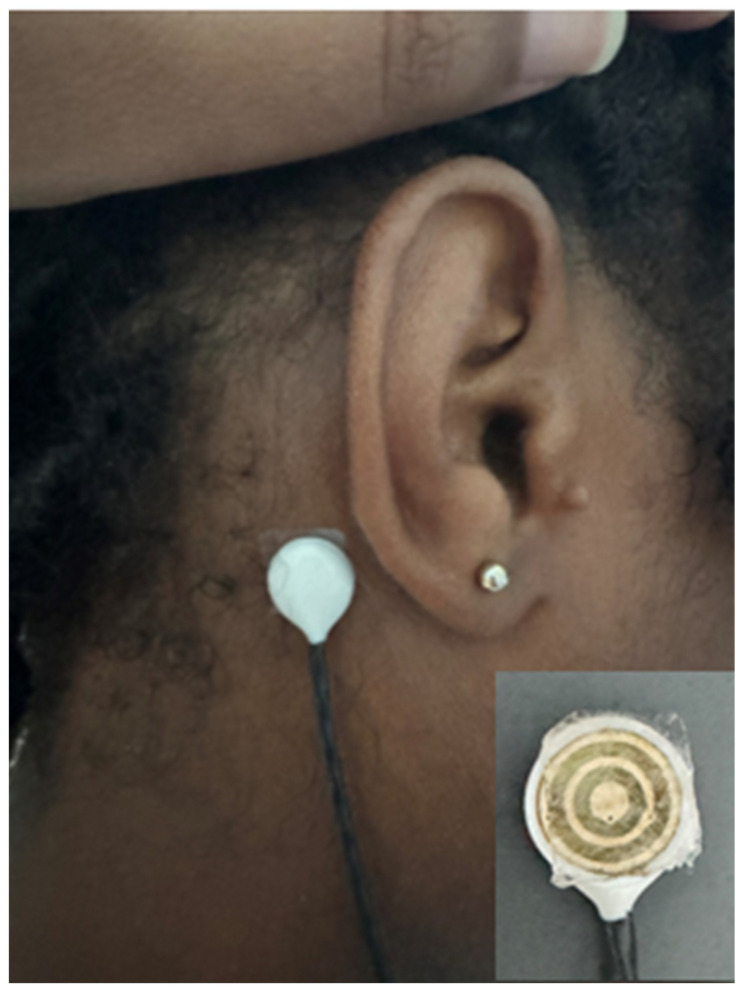
A TCRE has been placed on the right mastoid process and held in place with the KM40C hydrogel tape. The inset in the lower right shows the KM40C hydrogel tape on the TCRE.

To ensure optimal contact and signal quality, a sanitizing wipe was employed to cleanse the area behind each ear. Following this, a piece of hydrogel tape, measuring approximately half an inch by half an inch, was applied to the mastoid process. The application process involved peeling off the first non-stick side, adhering the tape to the mastoid process, and applying pressure for 10 s. Subsequently, the second non-stick side was removed, and a TCRE was affixed to the tape, employing a 10 s pressure application. This sequence was then replicated on the opposite side using a different hydrogel tape. An impedance meter was used again to measure between each tape behind the ear and the ground electrode. To ensure balanced testing, each of the four hydrogel tapes was randomly assigned to either the right or left mastoid process on each participant. The placement was alternated for subsequent sessions, ensuring that each tape was tested on both sides at least twice over the course of the study involving the 10 participants. Figure 2 shows the placement of the TCRE on the tape and the right mastoid process. The inset of Figure 2 shows the KM40C hydrogel tape on a TCRE.

### 2.6. Hardware Used for Data Collection

To record data, two TCREs were connected to a t-Interface 2 CH preamplifier (CREmedical, East Greenwich, RI, USA) which was then connected to a V-Amp (Brain Products, Gilching, Germany) using a ribbon cable with touch-proof connectors. The data were recorded using Recorder (Brain Products) with no filters used. The sampling rate was 1000 samples per second. Nine channels were recorded in total. There were four channels recorded for each TCRE. Channels 1–4 were for TCRE 1 and channels 5–8 were for TCRE 2. Channels 1 and 5 had a gain of 187 and were used for tEEG. Channels 2 and 6 had a gain of 1 used for eEEG. Channels 3 and 7, tEEG 5V, had a gain of 22,627. Channels 4 and 8, eEEG 5V, had a gain of 1331. Channel 9 was from a conventional disc electrode at the Oz location and connected directly to the Vamp. The V-Amp also possesses a gain of 1000, but as with other EEG amplifiers, that was removed in the acquisition software.

### 2.7. Data Collection

**Figure 3:**
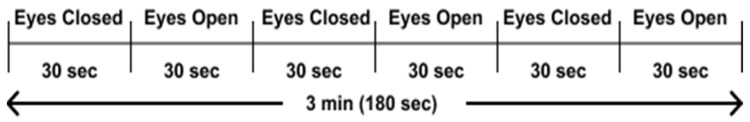
Schematic of the experimental paradigm timing. This procedure was repeated four times for the four different hydrogel tapes.

When the eyes are closed, many people generate a brain wave between 8 and 12 Hz that is dominant in the occipital areas [17]. It is typical when testing hydrogel tapes to use the eyes open or closed paradigm and perform a power spectral density function to compare the EEG during those times [13,18,19,20]. Other researchers also measure the skin-to-electrode impedance to help characterize the contact impedance. These researchers made disc electrodes of conductive hydrogels and found that they made good contact, were stable over the times recorded, and provided similar signals to conventional pastes [13,18,19,20]. For a simple disc electrode, they prefer high conductivity. That is not the case for a complex TCRE sensor. However, we followed a similar procedure as they reported that allowed us to have a common signal to compare the tapes with. During the EEG recording sessions, the participants were instructed to sit in a comfortable armchair and alternately close and open their eyes in a controlled manner, with each condition lasting 30 s (Figure 3). This procedure was repeated continuously for a duration of 3 min. The hydrogel tapes were applied randomly to prevent potential order effects.

### 2.8. Signal Processing

To process the data, we used a custom app designed by one of the members of our Neural Rehabilitation Laboratory at the University of Rhode Island that runs in EEGLab [21], an extension for Matlab version 2024A (Mathworks, Natick, MA, USA). The script the app ran was designed to search for peaks in a frequency range of the power spectrum we chose. For this study, a frequency range centered at 10 Hz was used since we were interested in alpha wave activity. The app utilized nine distinct channels and the corresponding saved marker events to analyze the neural activity of participants. Channels one through four were dedicated to amplifying the signals from a TCRE on the left mastoid from one hydrogel tape. Channels five through eight were from a second TCRE on the right mastoid and a different hydrogel tape. The ninth channel was a signal from a disc electrode over the occipital lobe, a typical location to capture strong alpha waves as a reference signal. 

The event sections, 30 s intervals, were denoted by comment markers entered manually into the Recorder application during which time the participants either had their eyes closed or open. For each section, session markers were placed in the recording to mark the tapes used, eyes open, and eyes closed. The eyes were closed between the closed–open markers. To extract peak power information, the data were processed by selecting the closed–open sequence. The app quantified the difference in peak power between the max peak and the baseline for each channel, Peak Diff. The baseline was the difference between base mean power from 12 Hz (the upper end of the alpha band, +3 Hz) to 45 Hz. Max peak was the maximum signal found between the frequency range of (sigp − 1) < sigp < (sigp + 1), where sigp was 10 Hz. The same analysis was systematically applied to all the channels, starting with closed–open and progressing to open–closed for each event. This iterative process continued until all the events were examined for each channel. 

To consolidate the findings, the Peak Diffs obtained during the eyes closed periods were averaged for each hydrogel tape, as were the Peak Diffs during the eyes open periods. The resulting average Peak Diffs during eyes open were subtracted from the average Peak Diffs during eyes closed.

(1) $$Avg\;{Peak\;Diff}_{C-O}={Avg\;Peak\;Diff}_{C}-{Avg\;Peak\;Diff}_{O}$$

This subtraction aimed to discern the presence of alpha waves despite the impedance introduced by the hydrogel tape. This entire analytical sequence was then replicated for each hydrogel tape, enabling a comprehensive exploration of alpha wave activity across different tapes.

### 2.9. Statistical Analysis

First, to determine if there was a significant difference in the signal power recorded with the eyes closed compared to when the eyes were open, a two-sample *t*-test assuming unequal variances was performed. The assumption here was that the eyes closed power should be greater than the power when the eyes were open; therefore, we used the one-tail *p*-value to determine significance. If there was no significant difference in the eyes closed power compared to the eyes open power, or if the eyes open power was greater than the eyes closed power, we assumed there were no alpha waves and did not perform any further analysis on that participant. A *p*-value of 0.05 was always used to represent a significant difference. To determine if the power during eyes closed was significantly different between the different conductive tapes, we used a single factor ANOVA on the Peak Diffs for the six instances for each participant. If there was a significant difference in the Peak Diffs power between the four tapes, we proceeded to perform a Tukey’s honest significance test, or Tukey’s HSD test, a single-step multiple comparison procedure and statistical test. This showed if there were any tapes that were always significantly different than the other tapes within a single participant. In this comparison, we assumed the tapes that registered the highest *Avg Peak Diff*_C-O_ were the best and the lowest *Avg Peak Diff*_C-O_ were not as good.

## 3. Results

### 3.1. Impedance of Hydrogel Tapes

The tripolar electrode has three rings: an outer, middle, and inner ring (Figure 1). We measured between these rings to determine which tapes had a high impedance or low impedance and would potentially impact the quality of the recordings. Table 1 shows that KM50L had the highest impedance of the tapes with a mean of 12.74 kΩ from outer to inner and 10.01 kΩ from middle to inner. Breachers possessed the lowest impedance of the four hydrogel tapes with a mean impedance of 3.72 kΩ between the outer and inner rings and 3.25 kΩ between the middle to inner.

### 3.2. Impedance between Hydrogel Tapes and Participants

The impedance was measured between the TCREs on the tapes after they were applied behind the ear and the ground electrode on the forehead. The impedance for all the tapes was large but stayed below 100 kΩ. Due to the higher impedance of the tapes, it was expected to see some signal attenuation in the recordings. Table 2 shows the impedance between the outer ring of the TCREs, which is behind the ear, and the ground electrode on the forehead. Channel 9 shows the impedance between the ground disc electrode and the second disc electrode placed above the external occipital protuberance.

### 3.3. Peak Power

Table 3 shows the average peak power for each tape while the eyes were closed, the alpha power. Channels 3 and 7 are for the high-gain tEEG and channels 4 and 8 are for the high-gain emulated EEG. From Table 3, we can see that the standard deviation was less for tEEG than eEEG. We can also observe that the power for the tEEG is higher than the eEEG power.

### 3.4. Signals Recorded

**Figure 4:**
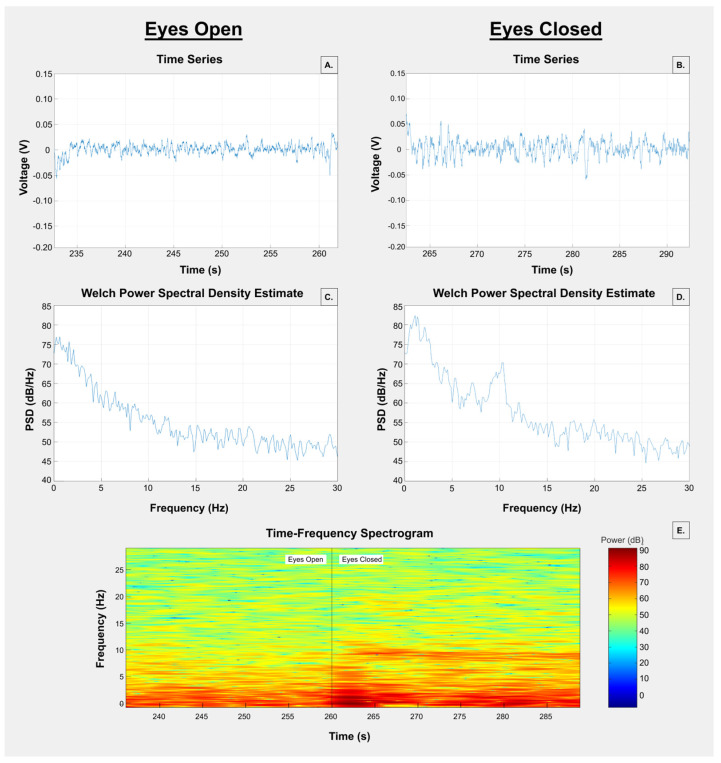
Upper row: tEEG measurements from participant C with the TCRE on the KM40C tape, recorded while eyes were open (**A**) and closed (**B**). Middle row: power spectrum, in dB, of eyes open (panel (**C**) from signals in panel (**A**)), and eyes closed (panel (**D**) from signals in panel (**B**)). Bottom, panel (**E**): spectrogram from the signals from panels (**A**) and (**B**). For panel (**E**), the vertical axis is the frequency that increases towards the top. The horizontal axis is time in seconds increasing to the right. The power intensity, in dB, is indicated with the color bar to the right of the spectrogram where dark red is the strongest power and dark blue is the weakest power.

Observing Figure 4, the top panels A and B show representative time series signals from participant C when eyes were open (panel A) and closed (panel B) with KM40C tape applied. All the signals shown are from TCRE 2 and recorded on channel 7 tEEG 5V with a gain of 22,627 from the right mastoid. The middle row, panel C, shows the power spectrum for the signals from panel A, and panel D is the power spectrum for the signals in panel B. Note that the power spectrum in panel D, from the signals while the eyes were closed, shows a clear peak around 10 Hz that is not seen in the power spectrum in panel C while the eyes were open. The bottom panel (E) is a spectrogram of the signals from panels A and B combined which are from TCRE 2, channel 7 tEEG 5V. For panel E, time is increasing to the right, frequency is increasing upward in the image, and the power is depicted by the color bar to the right. The spectrogram also shows a strong power component lasting nearly the entire segment of eyes closed at approximately 10 Hz.

### 3.5. Statistics

There were two participants, out of the ten, who did not have alpha waves. For them, there was no significant difference between the Peak Diffs of eyes open vs. eyes closed. For the other eight participants, there was at least one tape of the four that had a significant difference between eyes open and eyes closed. A single factor ANOVA for the eight participants who had alpha waves on the Peak Diffs for eyes closed was performed next. If there was a significant difference, then we proceeded to the Tukey HSD test. All eight participants had a significant difference. In five such comparisons, the KM50L tape was the outlier with a significantly lower Peak Diff than the other tapes. There were three instances for the KM40A tape when the significant difference was due to the Peak Diff being much higher than the other three tapes. A similar situation occurred twice each for the KM40C and KM50L tapes. The Breachers tape was never the worst (lowest Peak Diff) or the best (highest Peak Diff).

## 4. Discussion

We analyzed the use of conductive hydrogel tapes with TCREs to determine if brain signals could be recorded using the combination. For most of the participants, eight of the ten, we were able to register alpha waves from the conventional disc electrode over the occipital lobe and the TCREs attached with hydrogel tapes on the mastoids. However, there were two participants who did not register alpha waves from the conventional disc electrode on the occipital lobe or the TCREs on the mastoid process. This is why we also recorded from the conventional disc electrode using paste over the occipital lobe as a gold standard. Without the disc recording, we would not have known whether the hydrogel tapes did not work or if there was some other problem. Furthermore, as expected, there was no significant difference in the Peak Diffs between eyes open and eyes closed for those two participants.

The KM50L hydrogel tape had the lowest Peak Diff power in five of the comparisons that showed a significant difference. Observing Table 1, it can be seen that the KM50L has the highest impedance between the outer to inner and middle to inner impedance measurements on the bulk hydrogel tape. Although we need to have a high enough impedance between the rings and the central disc of the TCRE sensor, it appears that the 10.01 and 12.74 kΩ may be too high to register a strong signal from the scalp. Conversely, from Table 1, the Breachers tape had the lowest impedance between the outer to inner and middle to inner, 3.72 and 3.25 kΩ, respectively. From the statistical analysis, there was no instance where the Breachers hydrogel tape had the highest or lowest Peak Diffs. This seems to suggest that the Breachers tape is on the lower-end impedance that could be used to register signals with the TCRE sensors. It can also be seen from Table 1 that the KM40C (6.17, 5.18 kΩ) and KM40A (4.07, 3.62 kΩ) impedances fall in the middle of the other two tape impedances. For the KM40C and KM40A tapes, there were two and three instances, respectively, where the Peak Diffs were significantly higher than the other tapes. This leads us to believe that there may be an impedance between the KM40C and KM40A impedance that works even better. However, we will not know until we can find a hydrogel tape with an impedance that fits that criterion.

There were several issues encountered in this study related to the adhesion of the hydrogel tapes to the skin. Despite following our procedure for skin preparation, tape application, and pressure application both initially and during electrode placement, consistent adhesion proved problematic towards the end of the experiment. The primary issue was the tapes’ inability to maintain adherence, leading to tapes either completely detaching from the skin or partially lifting, resulting in sections of the tape hanging off the participants. These adhesion failures could be attributed to several factors, including variations in skin condition among the participants, the potential degradation of adhesive properties over time due to exposure to air or skin oils, and possible inconsistencies in the pressure applied during the attachment process. Furthermore, environmental factors such as humidity and temperature could have influenced the adhesive performance of the tapes. Future work with these tapes would need to consider ways to improve the adhesiveness. This may be managed by getting commercial hydrogel tapes that are tackier and possibly by using larger patches of the hydrogel tapes. Additionally, the tapes should achieve better uniform adhesion between the TCRE disc and the scalp, as this would enhance the accuracy and consistency of the experimental results.

Although using conductive hydrogel tapes would be convenient, they would not be practical in locations where there is hair. We found that we were able to record alpha waves with all four conductive hydrogel tapes. However, there were multiple sessions when the KM50L tape had significantly lower alpha wave power than the other tapes. The KM50L tape was the only conductive hydrogel tape that had significantly lower alpha wave power than the other tapes. We believe that using hydrogel tapes offers a promising solution for areas not covered with hair by providing a convenient, mess-free application process while maintaining reliable electrode–skin contact throughout EEG recordings. Based on our results, we would recommend the KM40A conductive hydrogel tape first, followed by the KM40C conductive hydrogel tape.

There has been a growing interest in frontal EEG [22]. Multiple applications where recording from areas on the head that do not have hair are plausible. For instance, in epilepsy, frontal and frontotemporal epilepsies make up approximately 25 percent of the epilepsies [23]. The focal sensing and the high-frequency capabilities of the tEEG could be effective in the frontal areas using hydrogel tapes to attach the TCREs. Furthermore, frontotemporal dementia has been researched with EEG [24]. It has also been reported that frontal EEG may be helpful for the diagnosis and treatment of Alzheimer’s Disease [25,26]. It has previously been shown that auditory potentials can be acquired with electrodes placed around the ears [27,28]. These applications, and more, could benefit from the tEEG focality and high-frequency capabilities when applied on the areas of the head without hair using hydrogel tapes.

## 5. Conclusions

In conclusion, conductive hydrogel tapes can be used with TCREs to acquire brain signals. Our recommendation for the areas without hair would be to use KM40A or KM40C for recording brain signals with TCREs. It is not certain, but it seems likely that the Breachers tape has too low of an impedance and KM50L appears to have too high of an impedance for the size of the TCRE sensors used in this study. We have shown that it is feasible to use commercially available hydrogel tapes on the mastoid process with TCREs to obtain alpha waves. However, it is going to take more effort to determine the practicality of hydrogel tapes with TCREs.

## Figures and Tables

**Table 1:** Mean and standard deviation of the impedances between the electrodes of a TCRE on the tapes.

Tapes	TCRE Rings Measured between	Mean (KΩ)	SD
KM40C	Outer to Inner	6.17	0.302
	Middle to Inner	5.18	0.270
KM40A	Outer to Inner	4.07	0.295
	Middle to Inner	3.62	0.270
KM50L	Outer to Inner	12.74	0.883
	Middle to Inner	10.01	0.750
Breachers	Outer to Inner	3.72	0.123
	Middle to Inner	3.25	0.127

**Table 2:** Impedance (Z), in kΩ, between the forehead reference electrode and outer ring of TCRE on the tapes.

Participants	Ch 9	Tape	Z Tapes	Participants	Ch 9	Tapes	Z Tapes
A	12.2	Breachers	55.3	F	12	Breachers	41.7
		KM40C	29.3			KM40C	41.8
		KM50L	20			KM50L	62.4
		KM40A	36.4			KM40A	50.9
B	13.2	Breachers	33.1	G	11.3	Breachers	40.1
		KM40C	22.3			KM40C	56.3
		KM50L	36.2			KM50L	36.3
		KM40A	21.4			KM40A	68.2
C	10.4	Breachers	32.6	H	12.7	Breachers	50.6
		KM40C	37.2			KM40C	26.9
		KM50L	41.2			KM50L	33.5
		KM40A	47.8			KM40A	48.6
D	11.6	Breachers	93.5	I	8.1	Breachers	90.6
		KM40C	72.3			KM40C	37.4
		KM50L	53.4			KM50L	53.4
		KM40A	56.4			KM40A	50.6
E	11.4	Breachers	76.1	J	11.2	Breachers	45
		KM40C	86.3			KM40C	31.8
		KM50L	92.3			KM50L	33.4
		KM40A	31.4			KM40A	37.5

**Table 3:** Averaged peak power in dB.

Tapes:	Channels	Average	STD
KM50L	3 and 7	14.1	3.44
	4 and 8	12.4	5.28
Breachers	3 and 7	14.7	2.59
	4 and 8	12.2	3.35
KM40C	3 and 7	15.0	6.03
	4 and 8	14.0	6.90
KM40A	3 and 7	16.8	2.40
	4 and 8	14.2	4.42

## Data Availability

Data are contained within the article and may be provided upon request.

## References

[1] Jacobs J., LeVan P., Chander R., Hall J., Dubeau F., Gotman J. (2008). Interictal high-frequency oscillations (80–500 Hz) are an indicator of seizure onset areas independent of spikes in the human epileptic brain. Epilepsia.

[2] Nunez P., Silberstein R., Cadusch P., Wijesinghe R., Westdorp A., Srinivasan R. (1994). A theoretical and experimental study of high resolution EEG based on surface Laplacians and cortical imaging. Electroencephalogr. Clin. Neurophysiol..

[3] He B. (1999). Brain electric source imaging: Scalp Laplacian mapping and cortical imaging. Crit. Rev. Biomed. Eng..

[4] Nunez P.L., Srinivasan R. (2006). Electric Fields of the Brain: The Neurophysics of EEG.

[5] LeVan P., Urrestarazu E., Gotman J. (2006). A system for automatic artifact removal in ictal scalp EEG based on independent component analysis and Bayesian classification. Clin. Neurophysiol..

[6] Besio W.G., Koka K., Aakula R., Dai W. (2006). Tri-polar concentric ring electrode development for laplacian electroencephalography. IEEE Trans. Biomed. Eng..

[7] Besio W.G., Cao H., Zhou P. (2008). Application of tripolar concentric electrodes and prefeature selection algorithm for brain-computer interface. IEEE Trans. Neural Syst. Rehabil. Eng..

[8] Koka K., Besio W.G. (2007). Improvement of spatial selectivity and decrease of mutual information of tri-polar concentric ring electrodes. J. Neurosci. Methods.

[9] Liu X., Makeyev O., Besio W. (2020). Improved Spatial Resolution of Electroencephalogram Using Tripolar Concentric Ring Electrode Sensors. J. Sens..

[10] Toole C., Martinez-Juárez I.E., Gaitanis J.N., Blum A., Sunderam S., Ding L., DiCecco J., Besio W.G. (2019). Source localization of high-frequency activity in tripolar electroencephalography of patients with epilepsy. Epilepsy Behav..

[11] Makeyev O., Boudria Y., Zhu Z., Lennon T., Besio W. Emulating conventional disc electrode with the outer ring of the tripolar concentric ring electrode in phantom and human electroencephalogram data. Proceedings of the 2013 IEEE Signal Processing in Medicine and Biology Symposium, SPMB 2013.

[12] El Ters N.M., Mathur A.M., Jain S., Vesoulis Z.A., Zempel J.M. (2018). Long term electroencephalography in preterm neonates: Safety and quality of electrode types. Clin. Neurophysiol..

[13] Shen G., Gao K., Zhao N., Wang Z., Jiang C., Liu J. (2022). A Fully Flexible Hydrogel Electrode for Daily EEG Monitoring. IEEE Sens. J..

[14] Xue H., Wang D., Jin M., Gao H., Wang X., Xia L., Li D., Sun K., Wang H., Dong X. (2023). Hydrogel electrodes with conductive and substrate-adhesive layers for noninvasive long-term EEG acquisition. Microsyst. Nanoeng..

[15] Oribe S., Yoshida S., Kusama S., Osawa S.-I., Nakagawa A., Iwasaki M., Tominaga T., Nishizawa M. (2019). Hydrogel-Based Organic Subdural Electrode with High Conformability to Brain Surface. Sci. Rep..

[16] Green R.A., Baek S., Poole-Warren L.A., Martens P.J. (2010). Conducting polymer-hydrogels for medical electrode applications. Sci. Technol. Adv. Mater..

[17] Chaddad A., Wu Y., Kateb R., Bouridane A. (2023). Electroencephalography Signal Processing: A Comprehensive Review and Analysis of Methods and Techniques. Sensors.

[18] Wang C., Wang H., Wang B., Miyata H., Wang Y., Nayeem O.G., Kim J.J., Lee S., Yokota T., Onodera H. (2022). On-skin paintable biogel for long-term high-fidelity electroencephalogram recording. Sci. Adv..

[19] Wang F., Yang L., Sun Y., Cai Y., Xu X., Liu Z., Liu Q., Zhao H., Ma C., Liu J. (2023). A Nanoclay-Enhanced Hydrogel for Self-Adhesive Wearable Electrophysiology Electrodes with High Sensitivity and Stability. Gels.

[20] Hsieh J.-C., Li Y., Wang H., Perz M., Tang Q., Tang K.W.K., Pyatnitskiy I., Reyes R., Ding H., Wang H. (2022). Design of hydrogel-based wearable EEG electrodes for medical applications. J. Mater. Chem. B.

[21] Delorme A., Makeig S. (2004). EEGLAB: An open source toolbox for analysis of single-trial EEG dynamics including independent component analysis. J. Neurosci. Methods.

[22] Gao Z., Cui X., Wan W., Qin Z., Gu Z. (2022). Signal Quality Investigation of a New Wearable Frontal Lobe EEG Device. Sensors.

[23] Beleza P., Pinho J. (2011). Frontal lobe epilepsy. J. Clin. Neurosci..

[24] Antonioni A., Raho E.M., Lopriore P., Pace A.P., Latino R.R., Assogna M., Mancuso M., Gragnaniello D., Granieri E., Pugliatti M. (2023). Frontotemporal Dementia, Where Do We Stand? A Narrative Review. Int. J. Mol. Sci..

[25] Adaikkan C., Middleton S.J., Marco A., Pao P.-C., Mathys H., Kim D.N.-W., Gao F., Young J.Z., Suk H.-J., Boyden E.S. (2019). Gamma Entrainment Binds Higher Order Brain Regions and Offers Neuroprotection. Neuron.

[26] Fatemi S.N., Aghajan H., Vahabi Z., Afzal A., Sedghizadeh M.J. (2022). Behavior of olfactory-related frontal lobe oscillations in Alzheimer’s disease and MCI: A pilot study. Int. J. Psychophysiol..

[27] Pacharra M., Debener S., Wascher E. (2017). Concealed Around-the-Ear EEG Captures Cognitive Processing in a Visual Simon Task. Front. Hum. Neurosci..

[28] Garrett M., Debener S., Verhulst S. (2019). Acquisition of Subcortical Auditory Potentials with Around-the-Ear cEEGrid Technology in Normal and Hearing Impaired Listeners. Front. Neurosci..

